# A systematic review and meta-analysis of postmastectomy radiation therapy on prepectoral versus subpectoral breast reconstruction

**DOI:** 10.3389/fsurg.2022.1019950

**Published:** 2023-01-09

**Authors:** Caihong Zheng, Jiameng Liu, Yahui Wen, Shunguo Lin, Hui Han, Chunsen Xu

**Affiliations:** ^1^ The Graduate School of Fujian Medical University, Fuzhou, China; ^2^ Department of Breast Surgery, Fujian Medical University Union Hospital, Fuzhou, China; ^3^ Department of General Surgery, Fujian Medical University Union Hospital, Fuzhou, China; ^4^ Department of Breast Surgery, Women and Children's Hospital, School of Medicine, Xiamen University, Xiamen, China; ^5^ Breast Cancer Institute, Fujian Medical University, Fuzhou, China

**Keywords:** breast, reconstration, postmastectomy, radiation, prepectoral, subspectral

## Abstract

**Background:**

Prepectoral breast reconstruction has once again appealed, which attributes to the introduction of acellular dermal matrices (ADMs) and mesh. Postmastectomy radiation therapy (PMRT), meanwhile, is crucial in the whole course of treatment for breast cancer patients with lymph node-positive. The impact of PMRT on outcomes after prepectoral breast reconstruction has not been clearly defined to date. This study aimed to compare the impact of PMRT on outcomes after prepectoral vs. subpectoral breast reconstruction.

**Methods:**

A comprehensive research on databases including PubMed, Embase, and Cochrane libraries was performed to retrieve literature pertaining to prepectoral breast reconstruction from database inception to October 2021. All included studies evaluated the impact of PMRT on outcomes after breast reconstruction. Only studies comparing patients who underwent prepectoral breast reconstruction with a control group who underwent subpectoral breast reconstruction were included. Data were analyzed using RevMan version 5.2.

**Results:**

A total of 4 studies were included in the meta-analysis, with a total of 394 breasts. In the setting of postmastectomy radiation therapy, 164 breasts were reconstructed with a prepectoral approach, whereas the remaining 230 breasts underwent subpectoral reconstruction. Overall, outcomes between PBR and SBR was no statistical significance in the overall complications (OR: 1.30, 95% CI: 0.35–4.85), infection (OR: 1.62, 95% CI: 0.90–2.91), seroma (OR: 1.60, 95% CI: 0.48–5.27), skin flap necrosis (OR: 0.77, 95% CI: 0.17–3.45), hematoma (OR: 0.38, 95% CI: 0.10–1.41), wound dehiscence (OR: 0.82, 95% CI: 0.36–1.85). But, included studies lacked data about the patient quality of life and satisfaction with the outcome of the reconstructed breast.

**Conclusions:**

In the setting of postmastectomy radiation therapy, prepectoral breast reconstruction is a safe and effective option.

## Introduction

Breast cancer is the most common malignancy in women around the world, subpectoral breast reconstruction had both demonstrated low complication rates and proved successful reconstructive modalities when utilized appropriately (1). Meanwhile, prepectoral breast reconstruction (PBR) is another approach that was abandoned by previous surgeons due to a lack of adequate soft tissue resulting in an unacceptably high complication rate (2). The introduction of acellular dermal matrices (ADMs) and mesh in 2006 reopened the possibility of prepectoral breast reconstruction (PBR) by harnessing the benefits of partial muscle coverage for improved breast projection and by providing inferolateral tissue support (3). However, in the setting of prepectoral breast reconstruction, where acellular dermal matrices (ADMs) and mesh provide the entire soft-tissue envelope immediately covering the implant, the outcomes in the setting of postmastectomy radiation therapy must be closely assessed and further studied.

Postmastectomy radiation therapy (PMRT) is an integral part of comprehensive treatment for breast cancer, due to its proven capacity to decrease local recurrence and improve survival (4). Findings also have shown that PMRT after breast reconstruction is associated with a higher rate of revisional surgery and worse cosmetic outcome as well as lower patient satisfaction (5). Recently, Hani's study (6) showed that there were no significant differences in individual complication rates between prepectoral and subpectoral groups in the setting of postmastectomy radiation therapy. But, from Thuman's (7) perspective, an increased percentage of complication rates in prepectoral breast reconstruction, but less severe complications and far fewer reconstructive failure compared with the subpectoral group.

Since the lack of randomized and prospective studies about the impact of PMRT on outcomes after prepectoral vs. subpectoral implant-based reconstruction and the limited number involved, the outcomes after reconstruction remain controversial. And there are few meta-analyses of the results of the impact of PMRT on outcomes after prepectoral vs. subpectoral breast reconstruction.

The purpose of this systematic review and meta-analysis is to evaluate the impact of PMRT on outcomes after prepectoral vs. subpectoral breast reconstruction, and further assess the safety and applicability.

## Material and methods

### Search strategy and selection criteria

A systematic literature search of PubMed, EMBASE and Cochrane Library databases pertaining to prepectoral breast reconstruction was conducted in October 2021. Utilizing PRISMA guidelines, databases were searched in varying combinations of keywords “prepectoral”, “subcutaneous”, “subpectoral”, “breast reconstruction”, “prosthesis”, and “Implant”. CZ and CX independently screened the title and abstract of the article to determine whether the article meets the eligibility criteria. The inclusion criteria were as follows: (1)the article described breast reconstructions with an implant placed either prepectorally or subpectorally. (2)patients who received post-mastectomy radiation therapy. (3) patient-reported outcomes were complications after breast reconstruction. (4)the article described a randomized controlled trial, a retrospective cohort study. (5) publication was from database inception to October 2021. (6) the full text was available. The full text was independently reviewed by CZ and CX, and differences were discussed by CZ, CX, and SL to reach a consensus, and the references of selected articles were screened to determine other relevant papers. Since this is an analysis of previously published articles, participants' informed consent and ethical approval are not required.

Reviews, comments, letters to the editor, animal studies, conference papers, case reports, and non-English articles were excluded.

### Data collection and analysis

Data were independently extracted by CZ and CX, including authors, publication year, study design, number of subjects, age, and follow-up period, followed by a record of the type of reconstruction and reported complications for all patients. Filtered patients were divided into two groups: the PBR group and the SBR group in the setting of postmastectomy radiation therapy (PMRT).

All of the statistical analyses were performed using RevMan version 5.2. Values of *p* < 0.05 were considered to be statistically significant. To ensure a better overall understanding we have calculated the odds ratio (OR) in every single study (PMRT to SBR vs. PMRT to PBR) as well as the weighted average of the ORs with 95% confidence intervals. All *p* values and 95% confidence intervals (CIs) were 2-sided. Statistical heterogeneity was evaluated using the I^2^ test. Statistically, significant heterogeneity was defined as an *I*^2^statistic > 40%. If heterogeneity was observed, we used a random-effects model to reduce the impact of heterogeneity on the results. If heterogeneity was not observed, a fixed-effects model was used. For each meta-analysis endpoint, a visual assessment funnel plot was used to assess potential publication bias. When *p* > 0.05, there was no publication bias.

## Results

### Included studies

**Figure 1:**
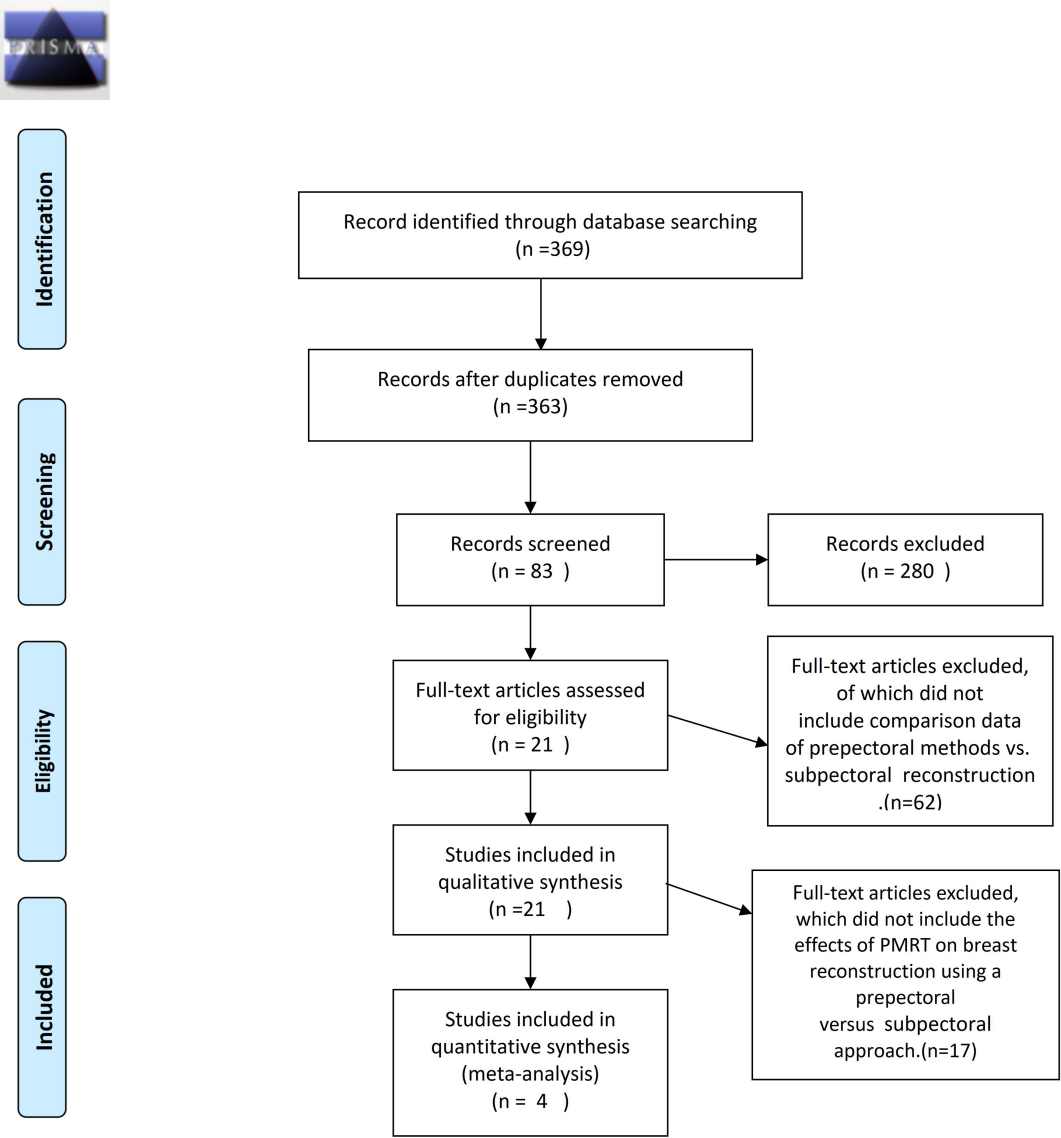
Flowchart demonstrating selection process for including studies. Meta-analysis data were collected following the PRISMA (preferred reporting items for systematic reviews and meta-analyses) guidelines.

A total of 369 publications were found through the database search published. Accumulated 369 papers were then filtrated first by title and then by the abstract of original articles pertaining to prepectoral breast reconstruction compared with subpectoral breast reconstruction resulting in 83 studies for full-text article assessment that were compared against inclusion criteria. Studies assessed the effect of surgical methods on breast reconstruction on the basis of PMRT, including extractable data. Finally, four studies (*n* = 394) were deemed eligible for meta-analysis (Figure 1).

### Meta-analysis

**Figure 2:**
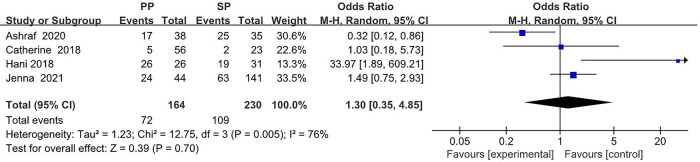
The meta analysis results of the overall complications.

**Figure 3:**
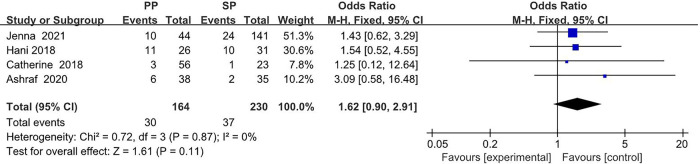
The meta analysis results of infection.

**Figure 4:**
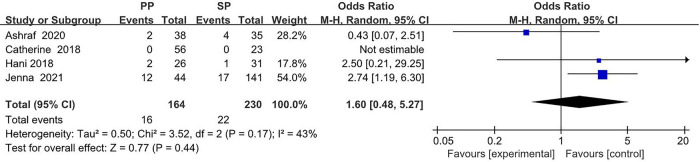
The meta analysis results of seroma.

**Figure 5:**
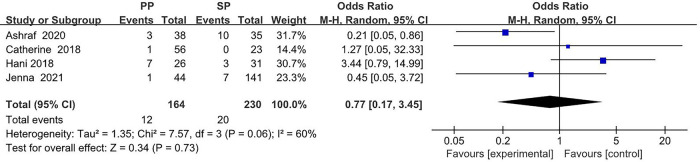
The meta analysis results of skin flap necrosis.

**Figure 6:**
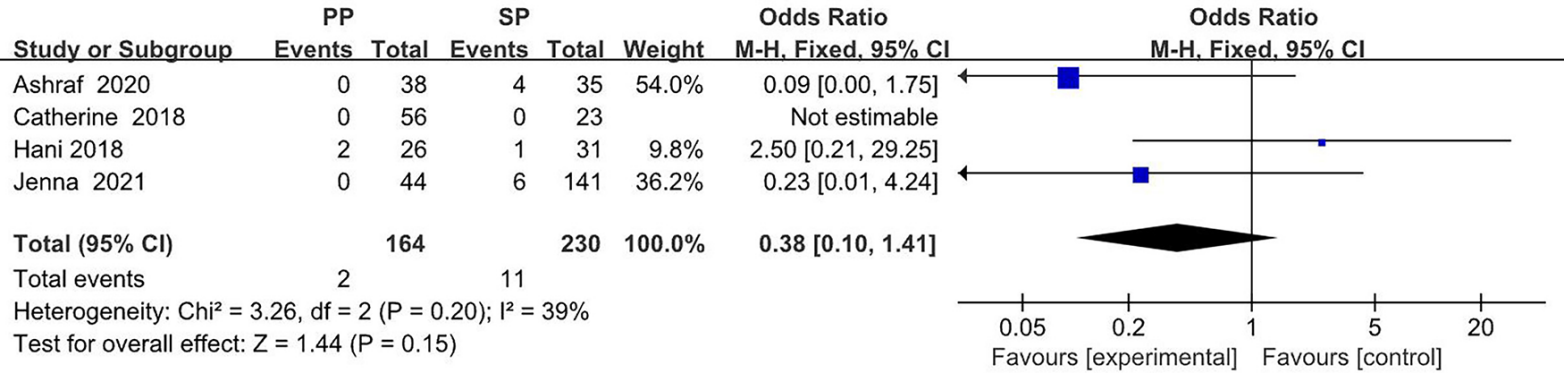
The meta analysis results of hematoma.

**Figure 7:**
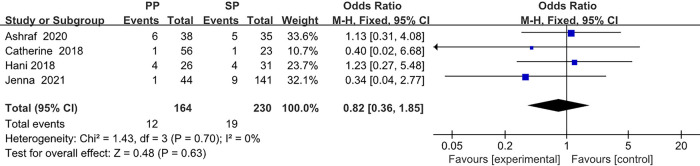
The meta analysis results of wound dehiscence.

The collected data from four studies (394 breasts) are summarized in Table 1. A total of 164 breasts in the setting of postmastectomy radiation therapy were reconstructed with prepectoral approach, whereas the remaining 230 breasts underwent subpectoral reconstruction. The meta analysis showed no statistical signification in the overall complications (OR: 1.30, 95% CI: 0.35 -4.85) (Figure 2), infection (OR: 1.62, 95% CI: 0.90 -2.91) (Figure 3), seroma (OR: 1.60, 95% CI: 0.48 -5.27) (Figure 4), skin flap necrosis (OR: 0.77, 95% CI: 0.17 -3.45) (Figure 5), hematoma (OR: 0.38, 95% CI: 0.10 -1.41) (Figure 6), wound dehiscence (OR: 0.82, 95% CI: 0.36 -1.85) (Figure 7). Finally, the whole funnel plots are presented. No publication bias exists.

**Table 1:** Complications of PMRT on breast reconstruction using a prepectoral vs. subpectoral approach.

Complications							
		Infection	Seroma	Hematoma	Necrosis	Dehiscence	Overall
Catherine et al.	PBR (56)	3	0	0	1	1	5
	SBR (23)	1	0	0	0	1	2
Hani et al.	PBR (26)	11	2	2	7	4	26
	SBR (31)	10	1	1	3	4	19
Jenna et al.	PBR (44)	10	12	0	1	1	24
	SBR (141)	24	17	6	7	9	63
Ashraf et al.	PBR (38)	6	2	0	3	6	17
	SBR (35)	2	4	4	10	5	25

### Infection

We performed a meta-analysis in two possible scenarios: a fixed-effect model or a random-effect model. In the ORs for infection, a relatively low level of heterogeneity among the included studies was found. Thus, the fixed effect model is mainly considered, which shows that 30 (18.3%) of 164 breasts in the PBR group and 37 (16.1%) of 230 patients in the SBR group were infected. There was no significant difference between the two groups (OR 1.62, 95% CI 0.90 -2.91, *I*^2 ^= 0%, *p *= 0.11) (Figure 3), indicating that prepectoral breast reconstruction did not increase the risk of postoperative infection in breast reconstruction patients.

### Seroma

For the ORs for seroma, high heterogeneity of *I*^2 ^= 43% was found. Thus, the random effect model is mainly considered, which shows that 16 (9.8%) of 164 breasts in the PBR group and 22 (9.6%) of 230 patients in the SBR group were infected. There was no significant difference between the two groups (OR 1.60, 95% CI 0.48 -5.27, *I*^2 ^= 43%, *p *= 0.44) (Figure 4), indicating that prepectoral breast reconstruction did not increase the risk of seroma in breast reconstruction patients.

### Skin flap necrosis

In the ORs for skin flap necrosis, high heterogeneity of *I*^2^ = 60% was found. Thus, the random effect model is mainly considered, which shows that 12 (7.3%) of 164 breasts in the PBR group and 20 (8.7%) of 230 patients in the SBR group were infected. There was no significant difference between the two groups (OR 0.77, 95% CI 0.17 -3.45, *I*^2 ^= 60%, *p *= 0.73) (Figure 5), indicating that prepectoral breast reconstruction did not increase the risk of skin flap necrosis in breast reconstruction patients.

### Hematoma

In the ORs for hematoma, a low level of heterogeneity among the included studies was found. Thus, the fixed effect model is considered, which shows that 2 (1.2%) of 164 breasts in the PBR group and 11 (4.9%) of 230 patients in the SBR group were infected. There was no significant difference between the two groups (OR 0.38, 95% CI 0.10 -1.41, *I*^2 ^= 39%, *p *= 0.15) (Figure 6), indicating that prepectoral breast reconstruction did not increase the risk of postoperative hematoma in breast reconstruction patients.

### Wound dehiscence

In the ORs for wound dehiscence, a low level of heterogeneity among the included studies was found. Thus, the fixed effect model is considered, which shows that 12 (7.3%) of 164 breasts in the PBR group and 19 (8.3%) of 230 patients in the SBR group were infected. There was no significant difference between the two groups (OR 0.82, 95% CI 0.36 -1.85, *I*^2 ^= 0%, *p *= 0.63) (Figure 7), indicating that prepectoral breast reconstruction did not increase the risk of postoperative wound dehiscence in breast reconstruction patients.

No data about the patient quality of life and satisfaction with the overall outcome and breast among patients following breast reconstruction are available.

**Figure 8:**
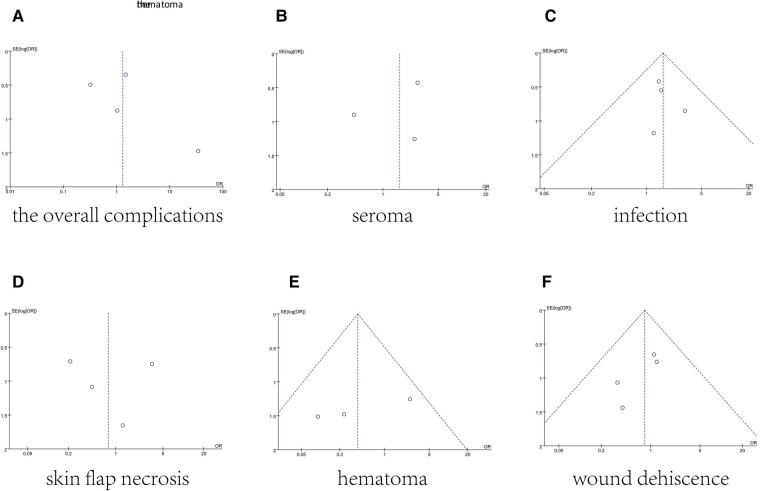
Funnel plots for the overall complications (**A**), seroma (**B**), infection (**C**), skin flap necrosis (**D**), hematoma (**E**), and wound dehiscence (**F**), which demonstrate no publication bias across included studies.

The funnel plot of total complications was shown in Figure 8. Mostly, points were inside the funnel from visual inspection. And Figure 8 provided funnel plots for each meta-analysis. In general, they had good symmetry indicating no evidence of publication bias (Figure 8).

## Discussion

Mastectomy results in loss of sensation in the skin of the breast and nipple-areolar complex, and loss of the breast for cosmetic, body image, and psychosocial health (8). All this has resulted in more women undergoing breast reconstruction. Meanwhile, for breast cancer patients with lymph node-positive, recommendations for radiation therapy for breast cancer in the postmastectomy setting are evolving as surgical modalities and systemic therapies improve (9).

Breast reconstruction in the setting of postmastectomy radiation therapy(PMRT) presents a unique challenge, because radiation therapy has an impact on the skin and soft tissues of the chest wall, leading to fibrosis that increases risks of contracture, infection, pain, necrosis, and atrophy in reconstructed tissues (10). Consequently, the discussion of the optimum operative method of breast reconstruction has not been stopped.

Traditionally, subpectoral implant-based breast reconstruction has remained the mainstay treatment choice for the majority (11). This approach requires stripping the pectoralis major muscle sternocostal fibers from the attachment of the costal cartilage and the lower part of the sternum in order to place a tissue expander (TE) or implant under the muscle (2). In this fashion, the potential for visible or stark implant contours is minimized and the incidence of capsular contracture, and implant loss is reduced (12). Over time, some studies stated that the subpectoral breast reconstruction could induce higher rates of implant malposition, animation deformity, and increasing postoperative pain due to contraction of the muscle (13). In some instances, this dynamic deformity becomes severe enough to distract from an overall excellent cosmetic result (14).

In recent years, with the introduction of acellular dermal matrices (ADMs) and mesh (15), an increasing number of researchers have shown that prepectoral breast reconstruction(PBR) has been viable approach even has been the optimum reconstruction approach when utilized appropriately (16). The prepectoral approach, because it avoided muscle dissection or elevation, has resulted in improved aesthetic outcomes with a more natural appearance, decreased postoperative pain, elimination of animation deformity, and shorter operative time (17).

This study showed no statistical significance in the overall complications (OR 1.30, 95% CI 0.35 -4.85), infection (OR 1.62, 95% CI 0.90 -2.91), seroma (OR 1.60, 95% CI 0.48 -5.27), skin flap necrosis (OR 0.77, 95% CI 0.17 -3.45), hematoma (OR 0.38, 95% CI 0.10 -1.41), wound dehiscence (OR 0.82, 95% CI 0.36 -1.85). Recently, Patel et al. (18) found that rates of overall complication were similar between PBR and SBR breast reconstruction in patients who received PMRT in delayed-immediate autologous breast reconstruction. Similarly, in Sinnott's study (19), the outcomes between the two groups did not significantly differ concerning infection, seroma, hematoma, dehiscence, mastectomy flap necrosis, or implant loss. But the patients who required PMRT after SBR had a capsular contracture rate three times greater, with more severe grade 3 or 4 contractures, than the patients who required PMRT after prepectoral breast reconstruction. As such, we always make it a routine to perform prepectoral breast reconstruction as the first option, in safe candidates, in the setting of known need for post-mastectomy radiation therapy delivery.

The patient quality of life and satisfaction with the overall outcome and breast among patients following breast reconstruction also is one of the key evaluation indicators. Nonetheless, no relevant data are available in this systematic review and meta-analysis.

Our study is to compare the impact of PMRT on outcomes after prepectoral vs. subpectoral breast reconstruction based on a comprehensive search. However, our study has its limitations. First, the included studies were not randomized controlled trials, but retrospective comparative studies. The inclusion of patients with potential selection bias and imbalanced baseline may affect the results of the study. Some variables, such as mesh material and implant surface, could not be accurately allocated between the two groups. Second, the difference in follow-up time may lead to an inaccurate assessment of the incidence of complications. In addition, other factors that may influence overall reconstructive success need further research in the future.

## Conclusion

For women who plan to undergo breast reconstruction in the setting of postmastectomy radiation therapy (PMRT), prepectoral breast reconstruction (PBR) is a safe and effective option.

## Data Availability

The original contributions presented in the study are included in the article/Supplementary Material, further inquiries can be directed to the corresponding author/s.
